# Concordance of Hospital Ranks and Category Ratings Using the Current Technical Specification of US Hospital Star Ratings and Reasonable Alternative Specifications

**DOI:** 10.1001/jamahealthforum.2022.1006

**Published:** 2022-05-13

**Authors:** Matthew E. Barclay, Mary Dixon-Woods, Georgios Lyratzopoulos

**Affiliations:** 1 THIS Institute, Department of Public Health and Primary Care, School of Clinical Medicine, University of Cambridge, Cambridge, United Kingdom; 2 Epidemiology of Cancer Healthcare Outcomes, Department of Behavioural Science and Health, Institute of Epidemiology and Health Care, University College London, London, United Kingdom

## Abstract

**Importance:**

The Centers for Medicare & Medicaid Services (CMS) Hospital Compare star ratings are widely used summaries of hospital quality that can influence patient choice and organizational reputation.

**Objective:**

To identify the changes in hospital ratings and rankings associated with alternative methodological choices in the calculation of the 2021 CMS Hospital Compare star ratings.

**Design, Setting, and Participants:**

This cross-sectional study used publicly available 2021 Hospital Compare data for 3339 US hospitals from the October 2020 data release. Change in apparent hospital performance was assessed, arising when plausible alternatives to current methods are used for calculating star ratings in relation to individual measure standardization, domain derivation, and domain weighting. Three example changes were examined in detail, with more comprehensive changes considered using Monte Carlo simulation. Changes in centile of hospital ranks and in star rating overall were examined, as well as separately in CMS peer groupings defined by the number of reported quality domains.

**Main Outcomes and Measures:**

Proportion of hospitals receiving a different star rating under the alternative technical specifications than under the current (2021) CMS approach and mean absolute change in centile rank of hospitals under alternative technical specifications compared with the current (2021) CMS approach.

**Results:**

A total of 3339 US hospitals were included in the analysis. Of the specific changes to current specifications considered, the alternative method of standardization was associated with the most substantial changes, with 55.4% (95% CI, 53.7%-57.1%) of hospitals having their star rating reclassified. The change in domain weights was associated with the smallest differences, but even that resulted in reclassification of the star rating in approximately 1 in 4 (24.5%; 95% CI, 23.0%-26.0%) hospitals. In the simulation study, which covered a range of possible changes, on average half of hospitals (51.8%; IQR, 44.2%-59.7%) were assigned a different star rating from that produced by the current specification, usually into an adjacent star category.

**Conclusions and Relevance:**

In this cross-sectional study of data on US hospitals, CMS Hospital Compare star ratings were found to be highly sensitive to how performance ratings are calculated, demonstrating the need for transparent justification of the technical approaches used in calculating composite performance ratings.

## Key Points

**Question:**

How are methodological choices about the construction of the Centers for Medicare & Medicaid Services Hospital Compare star ratings associated with the quality ratings of US hospitals?

**Findings:**

In this cross-sectional study of data on 3339 US hospitals, on average half were assigned a different star rating when using reasonable alternatives to the current methods. Different changes to the technical specification had differential outcomes, but even minor changes in specification could lead to substantial reclassification between adjacent performance categories.

**Meaning:**

The Centers for Medicare & Medicaid Services Hospital Compare star ratings are very sensitive to methodological choices about how they are calculated, raising questions about their design and transparency.

## Teaser

This cross-sectional study aims to identify the changes in hospital ratings and rankings associated with alternative methodological choices in the calculation of the 2021 Centers for Medicare & Medicaid Services Hospital Compare star ratings.

## Introduction

A high-profile scheme for public reporting of hospital care quality, the Centers for Medicare & Medicaid Services (CMS) Hospital Compare star ratings system assigned a rating to 3339 US hospitals in 2021.^1^ The star ratings, where 1 star corresponds to worst care and 5 stars to best, are intended to support patient choice^2^ and are used in hospital marketing.^3,4,5^ Star ratings and their underlying composite scores are also used in research examining predictors of hospital care quality.^6,7,8^ The scheme makes detailed methods information and statistical code available,^9,10^ but concerns have been expressed about the decision process used to develop the star ratings indicators, their suitability for measuring the quality of different types of hospitals, and the validity of some of the underlying measures.^11,12,13,14,15^ It remains unclear what effect using alternative reasonable approaches to deriving the CMS star ratings might have on the ratings assigned to individual hospitals.

Designing composite quality ratings involves many technical decisions.^16^ They include, for example, decisions about design and selection of the underlying measures, how final ratings are assigned, and which hospitals are deemed comparable. In this cross-sectional study, we focus on 2 specific sets of technical decisions. The first relates to how hospital-level performance on individual measures is judged. Here, we examine the standardization of hospital-level scores on individual measures. The second set of decisions concerns the approach taken to combining measures into some overall summary of quality. Here, we examine technical choices made in the grouping of individual measures into domains of quality and the weights given to each domain of quality. Our aim was to describe the spread of hospital ranks and star ratings overall and by number of domains of quality contributing to these composite measures of performance, as well as to assess the sensitivity of ratings of hospital performance to the technical specifications used to derive the CMS star ratings, both overall and across hospital peer groupings defined by the number of quality domains reported by hospitals.

## Methods

### Study Design

We calculated baseline hospital-level performance (scores, ranks, and star categories) for the April 2021 star ratings using the methods used by CMS.^9,10^ We then varied the technical specifications using reasonable alternative methods and recalculated performance under these specifications (a summary of the technical approaches considered is described herein and in Table 1). These alternative star ratings were compared with those derived using the 2021 CMS specification, with the aim of characterizing the sensitivity of the reported performance ratings of individual hospitals to the technical definition used to derive them. The 2021 CMS specification assigns star ratings within hospital peer groups defined by the number of reported domains of quality (all 5, 4, or 3) reported by each hospital. We repeated the analysis across these 3 groups. Because this was an analysis of publicly available hospital-level performance data, ethics review was not required.

**Table 1.** Baseline (2021) Technical Specifications of How Star Ratings Are Derived by CMS, Alongside Their Possible Limitations, and Justification of Alternatives

Technical specification	Baseline (2021) CMS approach	Limitations of 2021 approach	Plausible alternative approach(es) considered in this study	Likely strengths of the alternative approach
1. Standardization of individual measures	z Scoring used to standardize individual measures so that standardized performance represents the number of standard deviations above or below the mean	Discards contextual information about each measure: certain differences in performance may appear similar after *z *scoring but may not be comparable in reality	Use of individual measure-specific rules for transforming hospital performance on each measure to a 0-100 scale	Choice of measure-specific rules allows for identification of “good” (as opposed to “above average”) care quality
2. Grouping of the 49 individual measures into higher-level domains	Individual measures are grouped into 5 domains, which align with those used by CMS to group measures	Measures in the same domain may be measuring different constructs, making interpretation more challenging because poor performance on some measures may be averaged out by better performance on other, uncorrelated measures grouped into the same domain	Assign measures to the same domain if they measure similar empirical constructs, using exploratory factor analysis	Makes it easier to understand domain scores because it avoids combining empirically unrelated measures
3. Weighting of different quality domains before combining them into the composite score	A weight of 0.22 is assigned to each of the 4 outcome domains and a weight of 0.12 to the single-process domain	The weights given to each domain lack justification; the outcome of choice of weights on hospital performance is unclear	Give each domain the same weight	Allows use of weight distributions in Monte Carlo simulation, which in turn allows for assessment of uncertainty owing to choice of weights, while treating the 2021 weights as the ones most likely to be optimal
			Draw plausible alternative weights from independent log-normal distributions centered on the 2021 domain weights	

Abbreviation: CMS, Centers for Medicare & Medicaid Services.

### Data Set and Alternative Technical Specifications

The 2021 CMS Hospital Compare star ratings scheme comprised 49 individual measures grouped into 5 quality domains (see eMethods 1 in the Supplement for a summary of the 2021 calculation of the CMS star ratings).^9^ We used the October 2020 Care Compare data.^1^ We examined 3 technical decisions involved in calculating CMS star ratings (Table 1): (1) how to standardize each individual measure, (2) how to group the individual measures into higher-level domains, and (3) how to weight different quality domains to combine into the composite score.

### How to Standardize Individual Measures

Standardization of the individual measures in the 2021 CMS specification uses *z* scoring^9^ such that each hospital’s score on each individual measure reflects the number of standard deviations the hospital’s performance differs from the performance of a typical hospital on the same measure. The approach ensures that performance on different measures is comparable but does not allow insight into the importance of any variability. For example, relative differences between hospitals may not offer much meaningful information about quality if the average performance is already very high or if there is little variation across organizations.

Using a scoring system linked to specific levels of performance on each individual measure is a reasonable alternative to a *z*-scoring approach. Scoring systems for each individual measure can be designed to account for the importance of meeting certain performance thresholds, and moving to such scoring rules might have an important effect on the ranking of specific hospitals. Therefore, we defined reasonable measure-specific standardization functions for each measure (eMethods 2 in the Supplement). One example is the measure of median time from emergency department arrival to departure. Mean (SD) observed performance in the examined data set was 151 (46) minutes. When using *z *scores, as in the baseline design of the CMS star ratings, a hospital with a median time from arrival to departure of 151 minutes would receive a score of 0 (on a scale from −3 to +3). The alternative standardization approach for this measure was based on the principle that there might be a theoretical minimum time required for assessment in the emergency department, for which it would be inappropriate to incentivize any shorter time, and a theoretical maximum time where most negative effects of a long delay have already occurred. Benchmarks were selected with reference to the observed range of performance on the measure, with median times of less than 120 minutes receiving a maximum score of 100 and score decreasing smoothly to near 0 for median times greater than 600 minutes (eMethods 2 in the Supplement). Under this alternative standardization, a time of 151 minutes gave a score of 99 out of 100.

### How to Group the 49 Individual Measures Into Higher-Level Domains

The CMS star ratings included 5 domains of quality in the 2021 specification a priori aligned with the formal CMS grouping of quality measures.^9^ Although this represents a reasonable design choice, it is possible that measures assigned to the same domain may not be logically related. A reasonable alternative approach is to assign individual measures to domains using exploratory factor analysis. This would have the benefit of ensuring that measures within domains are empirically related.^17^ We used this approach to group each of the 49 individual measures into 1 of 6 higher-level domains (eMethods 3 in the Supplement).

One example is the baseline safety of care domain. It includes 8 individual measures, including hip and knee surgery complications and central line–associated bloodstream infections. They are all intended to represent measures of hospital safety but seem to measure different and perhaps even unrelated aspects of care safety, leading to domain scores that are hard to interpret. Factor analysis identifies empirically related measures, where scores on the different measures tend to point in the same direction. Domain scores from empirically coherent domains are likely to be more interpretable but may not immediately relate to clinical concepts of care quality.

### How to Weigh Different Quality Domains When Combining Them Into the Overall Composite Score

The 2021 CMS specification for the Hospital Compare star ratings assigns a weight of 0.22 to the 4 outcome domains (mortality, safety, readmission, and patient experience) and a weight of 0.12 to the single-process domain (timely and effective care). Although the choice of these weights aligns with other quality initiatives,^9^ there is no uniformly accepted standard to justify their suitability. Many alternative sets of domain weights could be considered (eg, giving the same weight to each domain).

### Statistical Analysis

Initially, we examined changes in star ratings and in hospital ranks associated with each individual change from the 2021 CMS technical specification, giving 3 comparisons of current CMS specification vs CMS specification but with alternative standardization method, CMS specification but with alternative domains, and CMS specification but with equal domain weights. Results were described using mean absolute changes in centile ranks (within hospital peer groups) and by examining shifts in star ratings.

We then used Monte Carlo simulation, with 10 000 simulations, to explore changes in hospital performance when the 3 aspects of the technical specifications were varied simultaneously. This meant simultaneously considering:

- Alternative approaches to standardizing individual measures (the *z *score approach or 1 specific alternative absolute scoring system)
- Alternative approaches to grouping individual measures (the 5 CMS domains or 6 alternative domains derived using exploratory factor analysis)
- A wide range of alternative domain weights drawn from probability distributions centered around the baseline (2021) weights (see eMethods 4 in the Supplement for details of the distributions used).

The results of the Monte Carlo simulation were summarized using reclassification rates and absolute change in centile rank, as well as the proportion of Monte Carlo draws in which each hospital achieved each star rating. We report results both overall and within peer groups defined by the number of domains of quality each hospital reported.

All analysis were conducted using Stata, version 17 (StataCorp). The Strengthening the Reporting of Observational Studies in Epidemiology (STROBE) study checklist was used to guide reporting.

## Results

Under the 2021 CMS approach and using October 2020 data, of the 3339 hospitals assigned a rating, 369 (11.1%) received a 5-star rating, 805 (24.1%) received 4 stars, 1007 (30.2%) received 3 stars, 839 (25.1%) received 2 stars, and 319 (9.6%) received 1 star (Table 2). The distribution of hospital star ratings was similar across hospital peer groups defined by the number of reported domains of quality (5, 4, or 3 domains) (Table 3).

**Table 2.** Performance of Hospitals Under the Baseline (2021) CMS Approach to Assigning Star Ratings and Change in Performance Under Each of the 3 Alternative Technical Specifications Considered Separately

Technical specification	Baseline (2021) rating	Hospitals, No. (%)	Proportion of hospitals receiving each rating under this alternative design, %					Hospitals reclassified, % (95% CI)^a^	Absolute change in centile of ranks, mean (95% CI)^b^
			5 Stars	4 Stars	3 Stars	2 Stars	1 Star		
Alternative standardization (external reference)	Any	3339 (100)	15.3	31.4	31.5	17.4	4.4	55.4 (53.7-57.1)	15.4 (15.0-15.9)
	5 Stars	369 (11.1)	60.4	32.5	7.0	0.0	0.0	39.6 (34.7-44.6)	11.3 (9.9-12.7)
	4 Stars	805 (24.1)	25.3	49.4	23.1	1.9	0.2	50.6 (47.1-54.0)	16.7 (15.7-17.7)
	3 Stars	1007 (30.2)	7.6	37.8	44.4	9.4	0.7	55.6 (52.5-58.7)	17.7 (16.9-18.4)
	2 Stars	839 (25.1)	0.8	17.0	41.2	37.3	3.6	62.7 (59.4-65.9)	16.0 (15.0-16.9)
	1 Star	319 (9.6)	0.3	1.9	14.4	49.2	34.2	65.8 (60.5-70.8)	8.7 (7.4-10.0)
Alternative domain grouping (factor analysis)^c^	Any	3323 (100)	11.9	29.0	30.9	20.2	8.0	31.9 (30.3-33.5)	6.6 (6.4-6.9)
	5 Stars	368 (11.1)	85.1	14.9	0.0	0.0	0.0	14.9 (11.7-18.9)	2.7 (2.4-3.0)
	4 Stars	799 (24.0)	10.4	79.0	10.6	0.0	0.0	21.0 (18.3-24.0)	6.9 (6.5-7.3)
	3 Stars	1003 (30.2)	0.1	27.4	63.5	9.0	0.0	36.5 (33.6-39.5)	8.8 (8.4-9.3)
	2 Stars	835 (25.1)	0.0	0.4	36.0	56.8	6.8	43.2 (39.9-46.6)	7.0 (6.5-7.5)
	1 Star	318 (9.6)	0.0	0.0	0.9	33.3	65.7	34.3 (29.3-39.7)	2.6 (2.2-3.0)
Alternative domain weights (equal weights)	Any	3339 (100)	13.8	30.0	30.2	19.3	6.6	24.5 (23.0-26.0)	2.6 (2.5-2.7)
	5 Stars	369 (11.1)	98.1	1.9	0.0	0.0	0.0	1.9 (0.9-3.9)	0.9 (0.8-1.0)
	4 Stars	805 (24.1)	12.4	86.8	0.7	0.0	0.0	13.2 (11.0-15.7)	2.7 (2.6-2.9)
	3 Stars	1007 (30.2)	0.0	29.5	69.8	0.7	0.0	30.2 (27.4-33.1)	3.5 (3.3-3.7)
	2 Stars	839 (25.1)	0.0	0.0	35.8	64.2	0.0	35.8 (32.6-39.1)	2.7 (2.5-2.8)
	1 Star	319 (9.6)	0.0	0.0	0.0	31.3	68.7	31.3 (26.5-36.6)	0.9 (0.8-1.0)

Abbreviation: CMS, Centers for Medicare & Medicaid Services.

^a^ Wilson score method.

^b^ Normal approximation.

^c^ A total of 16 hospitals rated under the current methods could not be assigned scores under the alternative domain groupings owing to missing measure information.

**Table 3.** Summary of Changes in Hospital Rating and Centile Rank Associated With the Different Alternative Technical Specifications Considered in the Monte Carlo Simulation by Baseline (2021) Star Rating, Overall, and Hospital Peer Group, Defined by the Number of Domains of Quality Reported

Peer group	Baseline (2021) rating	Hospitals, No. (%)	Ratings across all Monte Carlo simulations, mean, %					Across Monte Carlo simulations, mean (IQR)	
			5 Stars	4 Stars	3 Stars	2 Stars	1 Star	Hospitals reclassified, %	Absolute change in centile of ranks
All hospitals	Any	3339 (100)	14.0	29.3	30.5	19.6	6.5	51.8 (44.2-59.7)	15.0 (11.7-17.7)
	5 Stars	369 (11.1)	60.8	31.3	6.5	1.3	0.1	39.2 (31.4-47.4)	11.2 (6.4-14.2)
	4 Stars	805 (24.1)	20.3	52.8	22.7	3.9	0.4	47.2 (40.4-55.0)	15.7 (12.3-18.7)
	3 Stars	1007 (30.2)	6.2	32.4	46.2	14.0	1.2	53.8 (46.7-60.9)	17.0 (14.6-19.5)
	2 Stars	839 (25.1)	2.0	12.0	36.5	42.2	7.3	57.8 (49.5-66.6)	15.5 (12.2-18.3)
	1 Star	319 (9.6)	0.6	3.2	12.7	39.1	44.3	55.7 (42.9-67.7)	9.8 (5.8-12.4)
All 5 domains reported	Any	2472 (100)	15.1	29.8	30.0	18.9	6.2	52.0 (44.7-59.8)	14.9 (11.9-17.4)
	5 Stars	263 (10.6)	65.5	29.1	4.6	0.7	0.1	34.5 (25.9-43.0)	10.6 (6.4-13.2)
	4 Stars	576 (23.3)	23.9	53.2	19.6	3.0	0.3	46.8 (41.1-54.0)	15.7 (12.5-18.5)
	3 Stars	769 (31.1)	6.6	34.6	45.3	12.6	0.9	54.7 (48.0-61.8)	17.0 (14.7-19.4)
	2 Stars	635 (25.7)	1.7	12.7	37.6	41.0	7.0	59.0 (50.1-68.3)	15.3 (12.3-17.9)
	1 Star	229 (9.3)	0.3	3.0	13.5	39.8	43.3	56.7 (43.2-69.4)	9.4 (5.9-11.6)
4 Domains reported	Any	767 (100)	10.9	28.2	31.9	21.5	7.4	50.1 (41.6-59.5)	14.8 (11.0-18.3)
	5 Stars	100 (13.0)	48.3	37.6	11.1	2.8	0.2	51.7 (36.0-68.0)	12.5 (6.4-16.8)
	4 Stars	209 (27.2)	11.1	52.1	30.6	5.7	0.4	47.9 (36.4-59.3)	15.4 (11.7-19.0)
	3 Stars	219 (28.6)	4.2	24.3	50.0	19.0	2.5	50.0 (41.1-58.9)	16.8 (14.0-20.0)
	2 Stars	166 (21.6)	1.5	8.7	32.0	48.6	9.1	51.4 (41.6-62.0)	15.2 (11.4-18.6)
	1 Star	73 (9.5)	0.6	2.6	9.9	38.6	48.3	51.7 (38.4-65.8)	9.4 (4.5-13.2)
3 Domains reported	Any	100 (100)	12.2	24.5	31.1	22.9	9.3	60.0 (47.0-74.0)	19.2 (10.8-27.3)
	5 Stars	6 (6.0)	60.3	23.2	12.6	3.7	0.3	39.7 (16.7-50.0)	14.1 (1.7-26.0)
	4 Stars	20 (20.0)	12.4	47.6	29.4	10.0	0.6	52.4 (30.0-75.0)	20.9 (8.4-32.0)
	3 Stars	19 (19.0)	12.6	32.9	39.2	14.1	1.2	60.8 (47.4-78.9)	18.3 (13.8-22.3)
	2 Stars	38 (38.0)	7.7	15.4	38.9	32.8	5.2	67.2 (55.3-81.6)	20.6 (13.6-27.4)
	1 Star	17 (17.0)	4.6	8.6	13.4	32.6	40.8	59.2 (35.3-76.5)	16.8 (5.9-26.9)

Applying each of the 3 main alternative specifications considered was associated with substantial change in the rated performance of hospitals when compared with the current (2021) CMS approach (Figure). When considering changes individually, the alternative standardization approach was associated with the greatest degree of reclassification of star ratings and overall changes in rank (Figure and Table 2). Specifically, the change in standardization was associated with a mean absolute change in centile rank of 15.4 (95% CI, 15.0-15.9) points and 55.4% (95% CI, 53.7%-57.1%) of hospitals having their star rating reclassified, with 9.9% (95% CI, 8.9%-10.9%) of hospitals being reclassified into nonadjacent star categories (eg, 4 and 5 stars would be considered adjacent but 4 and 2 stars would not). Change in standardization was the only individual change where more than 1% of hospitals were reclassified into nonadjacent star categories. The alternative specification of domain weights (to an equal weighting approach) was associated with the least difference, with an average absolute change in centile rank of 2.6 (95% CI, 2.5-2.7) points. Despite the small differences in centile rank, about a quarter (24.5%; 95% CI, 23.0%-26.0%) of hospitals had their star rating reclassified (usually into a neighboring category) under alternative domain weights.

**Figure.**
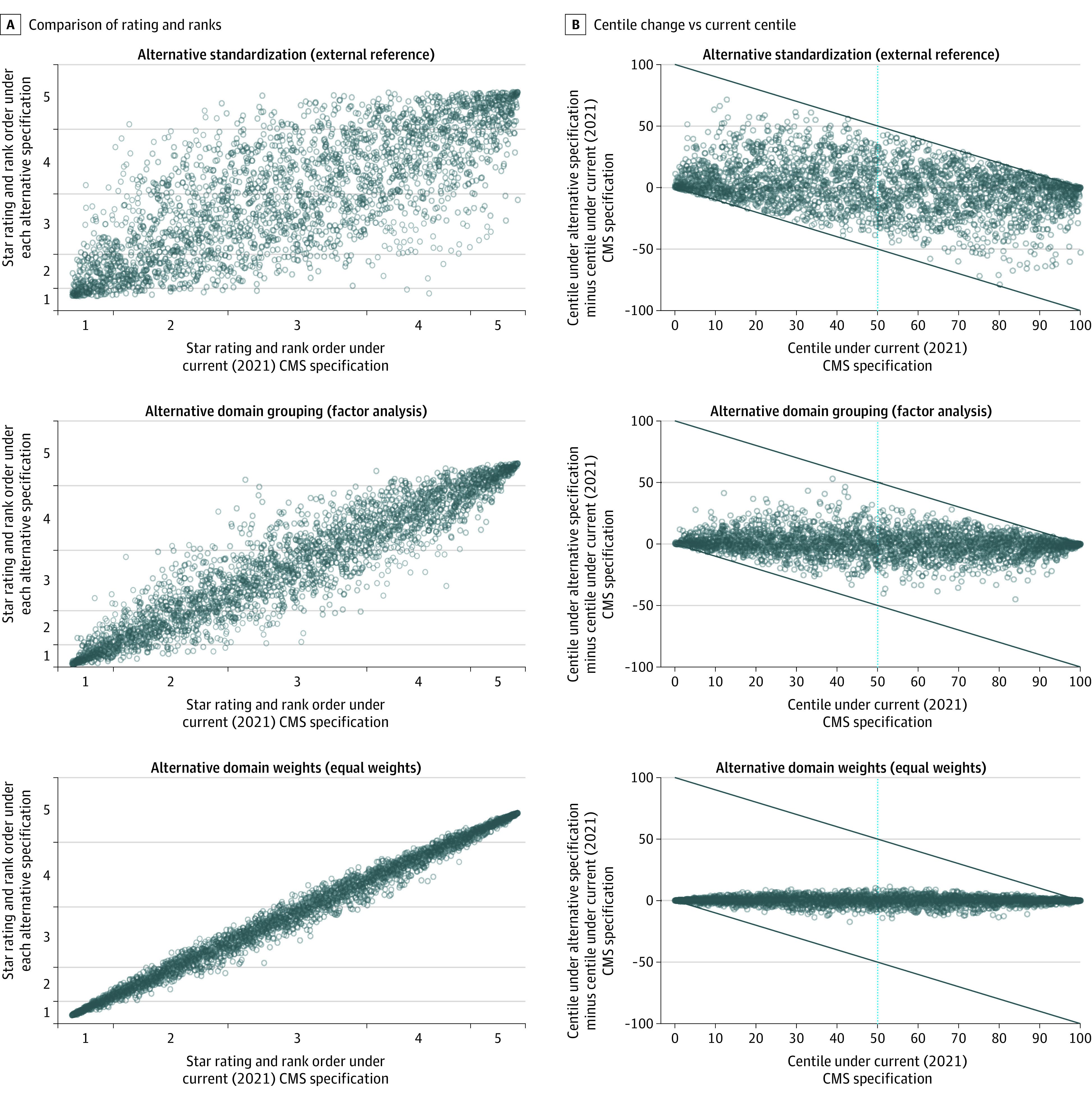
Differences in Hospital Ranks and Star Ratings Associated With the 3 Main Alternative Specifications Considered CMS indicates the Centers for Medicare & Medicaid Services.

In Monte Carlo simulations, the alternative technical specifications considered were associated with a typical hospital changing centile rank by a mean (IQR) of 15.0 (11.7-17.7) points (Table 3). On average, 39.2% (IQR, 31.4%-47.4%) of hospitals receiving 5 stars under the 2021 CMS approach dropped out of the category, and 7.9% (IQR, 1.9%-11.9%) would receive 3 or fewer stars. Overall, the different hospital peer groups (based on number of domains reported) appeared to have broadly similar sensitivity to the changes to the technical specification considered.

While reclassification between adjacent categories (eg, 4 stars to 5 stars or vice versa) was common following changes in technical specifications, more substantial changes were relatively rare (Table 3). For example, on average 79.0% (IQR, 74.3%-84.2%) of 4- or 5-star hospitals in the CMS approach were classified as 4 or 5 stars under alternative specifications in the Monte Carlo simulation, 46.2% (IQR, 39.1%-53.3%) of 3-star hospitals were classified as 3 stars, and 58.9% (IQR, 49.6%-69.2%) of 1- or 2-star hospitals were classified as 1 or 2 stars.

## Discussion

The frequently problematic nature of composite indicators of hospital quality is increasingly well recognized.^16^ The present findings show that CMS Hospital Compare star ratings are highly sensitive to methodological choices about their calculation. This means that using different but reasonable methods from those specified by CMS can be highly consequential. Many top-ranked hospitals under the 2021 CMS specification would lose their 5-star status under reasonable alternative approaches to standardizing individual measures, deriving quality domains, and domain weighting. Hospitals rated as 5 stars under the CMS specification were classified as 4-star hospitals under two-fifths of the alternative technical specifications we considered, while more extreme reclassifications were relatively rare. This suggests that differences between 4- and 5-star hospitals and 1- and 2-star hospitals might be indicative of real differences in performance, but differences between adjacent categories should not be assumed to be meaningful.

These findings suggest that relatively minor perturbations to underlying summary scores used in star ratings may lead to substantial numbers of hospitals being reclassified. The changes associated with shifting from the current (2021) CMS domain weights to equal weights, which a priori could be deemed a relatively minor change, are an example of this. On average, hospital ranks changed by just 2.6 centiles, yet 24% of hospitals received a different rating. The substantial amount of reclassification described in this study partly reflects inherent limitations in reporting performance using such ordinal categories: hospital performance may remain stable, but technical choices made in how ratings and rankings are calculated may easily influence their apparent, publicly reported performance.

### Context of the Literature

#### Examining the Design of Composite Indicators

Most previous analyses of the sensitivity of composite indicators to technical approaches have only assessed single dimensions of technical specifications,^18,19,20,21,22^ including previous examinations of the outcomes of alternative technical specifications of CMS star ratings.^23,24,25^ Detailed examinations of multiple technical aspects of composite indicators in the literature are rare. Exceptions include a 2004 discussion of multiple technical aspects of the construction of composite indicators in health care, based on hypothetical example and limited number of measures and not examining multiple technical choices simultaneously.^26^

#### Reporting of Composite Indicators

Most assessment of the effect of technical decisions on hospital performance on composite scores thus far has focused on the issues of weighting. Rumball-Smith and colleagues^27^ suggest providing “personalized hospital ratings,” where users can set their own domain weights to prioritize specific areas. While this is an appealing suggestion, the present findings illustrate that domain weights are not the only influential decision in producing composite indicators—and it is not necessarily easy to vary other important design aspects such as standardization or how measures are grouped into domains.

#### Justification of Decisions Taken When Developing Composite Indicators

Published sensitivity analyses of health care composite indicators are uncommon. Although it has been suggested that such analyses should be carried out,^28,29^ they are not typically reflected in public-facing documentation or publications. Justification of the decisions relating to the technical specifications adopted in the CMS star ratings, and a description of alternative approaches that may have been considered,^14,16^ does not appear to be publicly available. This study demonstrates the need for transparency about decisions relating to the development and specification of composite indicators. An example of such transparency is the US Baby-MONITOR composite indicator of neonatal intensive care unit quality, where developers compared 5 different approaches to weighting and aggregating measures, and justified their chosen approach based on the fact that all 5 approaches gave similar results.^30^

### Strengths and Limitations

We used a large sample of real-world performance data and examined multiple aspects of the technical design of the composite indicator. We demonstrated that design choices about technical specifications for calculating performance matter greatly for 2021 CMS star ratings for typical hospitals. We also described the sensitivity of hospital performance ratings within the CMS peer groups defined by the number of domains of quality reported.

Although we examined 3 reasonable different alternatives to the 2021 technical specification, these represent only a fraction of all of the technical choices involved in producing a large composite indicator like the star ratings. The decisions we examined as alternatives were reasonable, but other reasonable approaches are also possible.

## Conclusions

In this cross-sectional study of data on US hospitals, we demonstrated the sensitivity of hospital performance ratings and rankings to the adoption of reasonable alternative technical specifications. The design of composite indicators needs transparent justification. There may be multiple reasonable ways to construct a composite indicator such as a star rating, and changes in specifications can be associated with substantial swings in hospital ratings, particularly between adjacent categories, including between the coveted 5-star rating and the 4-star rating. This finding questions the extent to which these ratings should be relied on for purposes of patient choice, organizational reputation, or research. Consideration of reasonable alternative approaches and an explanation of why a chosen approach was preferred should form part of expected practice.

## Notes

Supplement. **eMethods 1.** Brief summary of the baseline (April 2021) calculation of the CMS Hospital Compare Star Ratings, as implemented for comparisons **eMethods 2.** Technical details of the alternative approaches to standardising individual measures **eMethods 3.** Technical details of the alternative approach to grouping measures into domains **eMethods 4.** Technical details of the alternative approaches to domain weights and the implementation in the Monte Carlo simulations
