# Supplementary material for: Concordance of Hospital Ranks and Category Ratings Using the Current Technical Specification of US Hospital Star Ratings and Reasonable Alternative Specifications
Source: JAMA Health Forum. 2022 May 13;3(5):e221006. doi: 10.1001/jamahealthforum.2022.1006 (PMC9107028; doi:10.1001/jamahealthforum.2022.1006)
Supplement: Supplement. — eMethods 1. Brief summary of the baseline (April 2021) calculation of the CMS Hospital Compare Star Ratings, as implemented for comparisons eMethods 2. Technical details of the alternative approaches to standardising individual measures eMethods 3. Technical details of the alternative approach to grouping measures into domains eMethods 4. Technical details of the alternative approaches to domain weights and the implementation in the Monte Carlo simulations [file jamahealthforum-e221006-s001.pdf]

# Supplemental Online Content

Barclay ME, Dixon-Woods M, Lyratzopoulos G. Concordance of hospital ranks and category ratings using the current technical specification of US hospital star ratings and reasonable alternative specifications. *JAMA Health Forum*. 2022;3(5):e221006. doi:10.1001/jamahealthforum.2022.1006

**eMethods 1.** Brief summary of the baseline (April 2021) calculation of the CMS Hospital Compare Star Ratings, as implemented for comparisons

**eMethods 2.** Technical details of the alternative approaches to standardising individual measures

**eMethods 3.** Technical details of the alternative approach to grouping measures into domains

**eMethods 4.** Technical details of the alternative approaches to domain weights and the implementation in the Monte Carlo simulations

This supplemental material has been provided by the authors to give readers additional information about their work.

© 2022 Barclay ME et al. *JAMA Health Forum.*

## eMethods 1. Brief summary of the baseline (April 2021) calculation of the CMS Hospital Compare Star Ratings, as implemented for comparisons

The technical specification of the CMS composite indicator is described in detail in their published technical methodology. This supplement summarises the baseline (April 2021) technical specification of the CMS Star Ratings.

CMS select 49 quality measures, which they group into five domains of quality (eMethods 1 Table 1). These five domains range in size from seven quality measures in the Mortality domain to 15 in the Timely and effective care domain. Each individual measure is mapped from its native scale (e.g. Percentages, rates or time-to-event) onto a common scale by Z-scoring using a normal approximation. Standardised scores (i.e. z-score values) greater than 3 are rounded down to 3, and any less than -3 are rounded up to -3, a process known as Winsorization. Applying Winsorization limits the influence any extreme scores may have on the overall performance of a hospitals. A score of +3 is the best possible and -3 the worst possible on every performance measure.

To estimate scores for each of the five domains of quality CMS take the arithmetic average of the individual measures reported by the hospital. The score is only calculated where hospitals report three or more measures.

The overall summary score is calculated by taking a weighted average of the various domain scores. Outcome domains are given a higher weight than the process domain (eMethods 1 Table 1). Hospitals receive a summary score if they report at least three of the five domains of quality.

Finally, CMS assign the overall star rating by applying $k$-means clustering to the overall summary scores, with $k = 5$. This splits hospitals into five groups such that each hospital is closer to the mean of its group than to the mean of any other group. The group with the highest score is classed as the ‘five star’ group, the next highest score is the ‘four star’ group, and so on.

To account for possible associations where hospitals with more missing data tend to receive higher scores, CMS assign hospitals to peer groups defined by the number of domains of quality for which they have scores. As hospitals must report at least three of the five domains to receive an overall summary score, this gives three groups: those that report all five domains; those that report four domains; and those that report three domains. The $k$-means clustering used to assign the star rating is carried out separately for each of these peer groups. Thus the score required to receive a five star rating, for example, may not be the same for all hospitals.

CMS prefer $k$-means clustering to other approaches because it provides a naturally defensible categorisation. With other approaches, such as splitting hospitals into five equal-sized groups, then there may be little distinction between performance categories. For example, if hospital performance followed a perfect normal distribution with mean 0 and standard deviation 1, then splitting into equal fifths leads to the middle performance groups covering only a narrow band of hospital scores (eMethods 1 Figure 1), while the top and bottom categories cover a very wide range of performances. Using $k$-means mitigates this problem while still guaranteeing five distinct performance categories.

© 2022 Barclay ME et al. *JAMA Health Forum.*

*eMethods 1 Table 1. Domains of quality and constituent measures used in the CMS Hospital Compare Star Ratings, with the proportion of hospitals with missing data on each individual measure.*

| Domain | Measure name | Measure type | % missing (all 3,339 hospitals) |
|---|---|---|---|
| **Outcome domains** | | | |
| Mortality | Death rate for heart attack patients | Proportion | 34.4% |
| | Death rate for CABG surgery patients | Proportion | 70.5% |
| | Death rate for COPD patients | Proportion | 3.6% |
| | Death rate for heart failure patients | Proportion | 2.2% |
| | Death rate for pneumonia patients | Proportion | 1.8% |
| | Death rate for stroke patients | Proportion | 27.4% |
| | Deaths among Patients with Serious Treatable Complications after Surgery | Proportion | 49.0% |
| Safety | Central line-associated bloodstream infections (CLABSI) | Rate ratio | 42.0% |
| | Catheter-associated urinary tract infections (CAUTI) | Rate ratio | 34.8% |
| | Surgical site infections from colon surgery (SSI: Colon) | Rate ratio | 45.2% |
| | Surgical site infections from abdominal hysterectomy (SSI: Hysterectomy) | Rate ratio | 75.5% |
| | MRSA bloodstream infections | Rate ratio | 49.1% |
| | C. diff bloodstream infections | Rate ratio | 12.6% |
| | Rate of complications for hip/knee replacement patients | Proportion | 25.1% |
| | Serious complications (PSI 90) | Rate | 14.7% |
| Readmission | Acute Myocardial Infarction (AMI) 30-Day Readmission Rate | Rate | 39.8% |
| | Rate of readmission for CABG | Rate | 70.8% |
| | Rate of readmission for chronic obstructive pulmonary disease (COPD) patients | Rate | 3.3% |
| | Heart failure (HF) 30-Day Readmission Rate | Rate | 2.2% |
| | Rate of readmission after hip/knee replacement | Rate | 25.1% |
| | Pneumonia (PN) 30-Day Readmission Rate | Rate | 1.9% |
| | Rate of readmission after discharge from hospital (hospital-wide) | Rate | 0.0% |
| | Rate of unplanned hospital visits after colonoscopy (per 1,000 colonoscopies) | Rate | 10.8% |
| | Rate of inpatient admissions for patients receiving outpatient chemotherapy | Rate | 54.5% |
| | Rate of emergency department (ED) visits for patients receiving outpatient chemo | Rate | 54.5% |
| | Ratio of unplanned hospital visits after hospital outpatient surgery | Rate | 18.4% |
| Patient experience | Patients who reported that their nurses communicated well | Proportion | 1.0% |
| | Patients who reported that their doctors communicated well | Proportion | 1.0% |
| | Patients who reported that they received help as soon as they wanted | Proportion | 2.7% |
| | Patients who reported that staff explained about medicines… | Proportion | 1.0% |
| | Cleanliness and quietness | Proportion | 1.0% |
| | Patients who reported that they were given information about what to do… | Proportion | 1.0% |
| | Patients who understood their care when they left the hospital | Proportion | 1.0% |
| | Rating and recommendation | Proportion | 1.0% |
| **Process domain** | | | |
| Timely and effective care | Percentage of healthcare workers given influenza vaccination | Proportion | 40.4% |
| | Percentage of patients who left the emergency department before being seen | Proportion | 7.1% |
| | Percentage of patients who came to the emergency department with stroke symptoms | Proportion | 53.7% |
| | Percentage of patients receiving appropriate recommendation for follow-up screen | Proportion | 20.7% |

© 2022 Barclay ME et al. *JAMA Health Forum.*

| Domain | Measure name | Measure type | % missing (all 3,339 hospitals) |
|---|---|---|---|
| | Percentage of patients with history of polyps receiving follow-up colonoscopy | Proportion | 21.1% |
| | Percentage of mothers whose deliveries were scheduled too early | Proportion | 30.1% |
| | Percentage of patients who received appropriate care for severe sepsis and septic shock | Proportion | 12.2% |
| | Percentage of outpatients with chest pain or possible heart attack who got drugs | Proportion | 97.5% |
| | Percentage of patients receiving appropriate radiation therapy for cancer that has spread to bones | Proportion | 75.5% |
| | Average (median) time patients spent in the emergency department, after the doctor had decided to admit | Time-to-event | 2.7% |
| | Average (median) number of minutes before outpatients with chest pain received specialist care | Time-to-event | 87.6% |
| | Average (median) time patients spent in the emergency department before leaving | Time-to-event | 3.9% |
| | % o/p with low-back pain who had MRI w/o trying recommended treatments first | Proportion | 62.1% |
| | % o/p CT scans of the abdomen that were combination (double) scans | Proportion | 2.9% |
| | % o/p who got cardiac imaging stress tests before low-risk outpatient surgery | Proportion | 38.6% |

© 2022 Barclay ME et al. *JAMA Health Forum.*

*eMethods 1 Figure 1. Illustrative comparison of categories assigned via $k$-means clustering and by splitting into fifths for a notional performance measure that follows a normal distribution with mean 0 and standard deviation 1.*

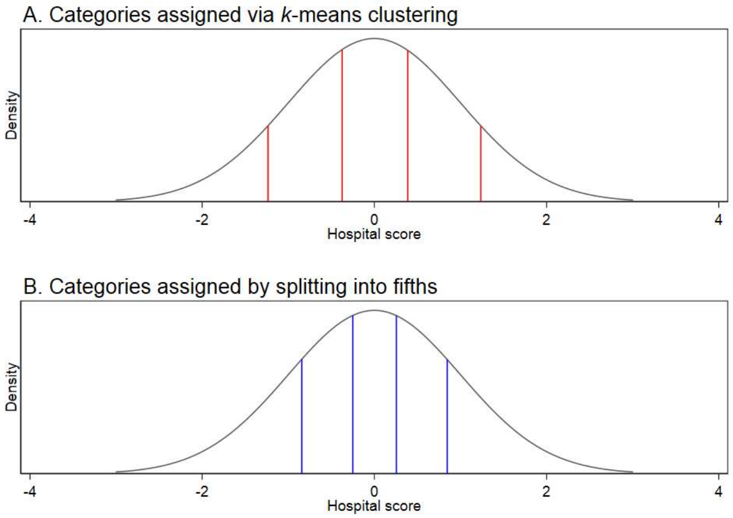


© 2022 Barclay ME et al. *JAMA Health Forum.*

## eMethods 2. Technical details of the alternative approaches to standardising individual measures

When standardising measures, the 2021 Star Ratings approach treats all individual measures that feed into each domain similarly, regardless of their nature, i.e. with regard to whether they constitute rates, proportions or time-to-event measures. The approach CMS used, known as 'Z-scoring', measures how different performance at a particular hospital is from the average relative to the differences observed across all hospitals in a particular year. If scores are normally distributed, then around 95% of hospitals will fall between ±1.96 standard deviations and 99.8% of hospitals between ±3.

In terms of the incentives provided to rated organisations, if performance is measured on a 1-100 scale it is desirable for a change in score from 2 to 1 to be of the same importance as a change in score from 62 to 61. Otherwise there is an incentive for organisations to focus improvement efforts in certain areas, and these areas may not be those that are most important. Z-scoring does not achieve this, and we defined alternative standardisation rules based on absolute performance on each measure that aimed to reflect the importance of particular levels of performance.

The Star Ratings include two different types of measures that might plausibly be treated differently. Of the 49 individual measures (see eMethods 1 Table 1), 46 are proportion and rate-based measures such as death or healthcare-acquired conditions. A further three measures represent time intervals, such as time in the emergency department from arrival to departure.

For the 46 proportion and rate-based measures, one could deem differences between higher and lower values to be equally meaningful whether the lower value is near zero or approaching 100% (or, for rate-based measures,100 events per 100 units of person-time). For example, a one-percentage-point reduction in post-operative mortality equates to one fewer death per 100 operations, whether it relates to a reduction from 2% to 1% or from 62% to 61%. But with Z-scoring a one percentage-point change will appear far more important for a performance measure with standard deviation of one percentage-point than for a performance measure with a standard deviation of three percentage-points. Hence, for these measures, Z-scoring distorts comparisons between different performance measures.

As a plausible alternative approach that avoids this distortion, we standardised all event (e.g. mortality) or rate (e.g. of healthcare acquired infection) measures according to the proportion of people having an event, so that differences between levels of do represented deaths avoided or safety events that did not happen. For example, a mortality rate of 0.18 would equate to a standardised score of 100*(1-0.18) = 82, while a mortality rate of 0.73 would equate to a score of 100*(1-0.73) = 27.

For the three of the 49 individual measures in the CMS Star Ratings representing time intervals, such as time in the emergency department from arrival to departure, a difference of one hour might be much more consequential if it is between, say, two and three hours than if it is between 15 and 16 hours. Z-scoring is not able to reflect the challenge that the impact of an additional hour of expected waiting time depends on how long the expected waiting time is. Hence, for these measures, Z-scoring risks distorting comparisons between different levels of performance on the same performance measure.

As a plausible alternative that reflected the variable importance of an additional period of waiting, we mapped time interval measures to the 0-100 scale using an appropriate logistic transformation: differences between middle-of-the-range performances were treated as more important than differences between excellent performances, or between poor performances.

© 2022 Barclay ME et al. *JAMA Health Forum.*

The choice of transformation depended on the measure, but was motivated by the idea that differences between groups of hospitals with either excellent (or very poor) levels of performance were not important, so they should be small on the standardised scale.

For example, for the time-to-event measure ‘ED – time arrival to departure’, our alternative approach to standardisation was motivated by the idea that hospitals with median intervals of two hours or less had excellent performance (independently of whether one hospital has a mean of 60’ and another of 90’ minutes), and therefore all deserved a top score. Conversely, hospitals with median scores of ten hours or more had very poor performance deserving of a minimal score (again, independently of between-hospital differences within hospitals comprising the 10+ hours group).

With these considerations in mind, we used the following function to standardise the time-to-event measure ‘ED – time arrival to departure’. Let $d$ be the standardised score for this measure and $t$ time from arrival in ED to departure in minutes. Then the standardised score $d$ is calculated as:

$$d = \begin{cases} 100 \text{ if } t < 120 \text{ minutes} \\ 100 \times \left(1 + \text{expit}(-5) - \text{expit}\left(-5 + \frac{t - 120}{48}\right)\right) \text{ if } t \geq 120 \text{ minutes} \end{cases}$$

This equation looks complex, but in practice it simply describes a smooth curve that falls slowly up to around four hours, then drops off rapidly until it levels off at around ten hours (eMethods 2 Figure 1A). There were 3208 hospitals with a known score for this performance measure, with mean performance 151 minutes (interquartile range 122 to 174 minutes). Mean standardised performance was 98.6 (interquartile range 98.6 to 99.97).

Standardised scores for the other four time-to-event measures were calculated as follows.

ED – time admit decision to departure (eMethods 2 Figure 1B). There were 3248 hospitals with a known score for this performance measure, with mean performance 110 minutes (interquartile range 64 to 135 minutes). Mean standardised performance was 76 (interquartile range 66 to 98).

$$d = \begin{cases} 100 \text{ if } t < 30 \text{ minutes} \\ 100 \times \left(1 + \text{expit}(-5) - \text{expit}\left(-5 + \frac{t - 30}{24}\right)\right) \text{ if } t \geq 30 \text{ minutes} \end{cases}$$

OP – time to specialist care (eMethods 2 Figure 1C). There were 415 hospitals with a known score for this performance measure, with mean performance 64 minutes (interquartile range 42 to 69 minutes). Mean standardised performance was 88 (interquartile range 92 to 99).

$$d = \begin{cases} 100 \text{ if } t < 30 \text{ minutes} \\ 100 \times \left(1 + \text{expit}(-5) - \text{expit}\left(-5 + \frac{t - 30}{15}\right)\right) \text{ if } t \geq 30 \text{ minutes} \end{cases}$$

© 2022 Barclay ME et al. *JAMA Health Forum.*

*eMethods 2 Figure 1. Comparison of observed and standardised scores under the plausible alternative standardisation approach for the five time-to-event measures used in the CMS Hospital Compare Star Ratings. The black line shows the standardisation function, and the blue circles show observed scores for individual hospitals.*

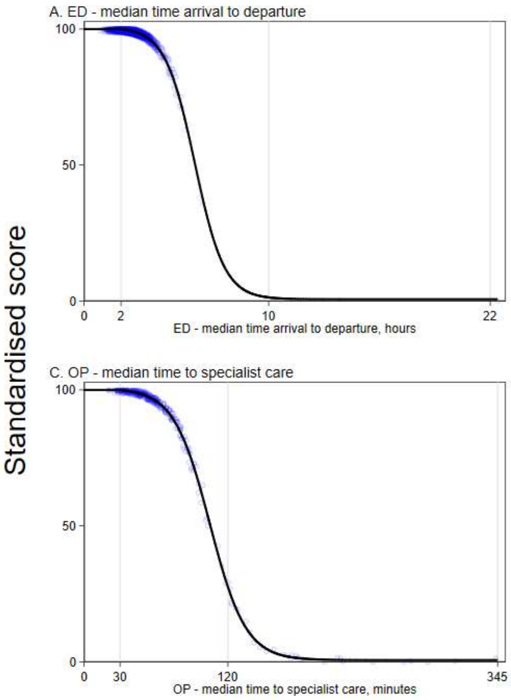


© 2022 Barclay ME et al. *JAMA Health Forum.*

## eMethods 3. Technical details of the alternative approach to grouping measures into domains

The baseline CMS approach assigns individual measures to five domains: four outcome domains (mortality, safety of care, readmission, and patient experience) and one process domains (timely and effective care) – see also eMethods 1 Table 1. These align with the domains used in the CMS Hospital Value-Based Purchasing program and other national quality initiatives.

As a plausible alternative approach, we used the hospital-level data on the individual measures to generate the quality domains, which were then combined into the overall composite score. We applied exploratory factor analysis to identify empirical latent factors that explained most of the variance among the individual measures.

Missing data presented a challenge to this alternative approach. Standard approaches to exploratory factor analysis require complete data on all measures for all hospitals, but all hospitals in the October 2020 dataset we used had some missing performance measure information (eMethods 1 Table 1). That is, for all hospitals there was at least one, and usually several, performance measures for which they had no performance information at all. The expectation-maximisation algorithm was used to estimate the covariance matrix between all measures,[1] giving correct results if measures were missing-at-random.[2]

The number of factors to retain was decided after inspecting scree plots and considering eigenvalues. While no strict criteria were applied, enough factors were retained to reach the 'elbow' of the scree plot, with this being interpreted generously so that most factors with eigenvalues above 1 were retained.

The promax rotation was applied to estimate how strongly each measure was associated with the underlying factors.[3] This process generated empirically coherent domains into which individual measures could be assigned. On the occasions where an individual measure was found to have a similar degree of association with more than one empirically-derived domain I assigned the measure to the domain where it appeared more conceptually relevant.

This factor analytic approach produced six empirical domains (eMethods 3 Table 1), rather than the five domains used in the April 2021 CMS Star Ratings. Some of these domains closely matched the baseline domains, with the patient experience domain being unchanged. Measures in other baseline domains were spread across multiple empirical domains (eMethods Figure 1). Details of the individual measures and the domains they were assigned to under the current approach and the factor analytic approach are in eMethods 3 Table 1.

*References*


1. Dempster AP, Laird NM, Rubin DB. Maximum Lifelihood from Incomplete Data via the EM Algorithm. *J R Stat Soc Ser B*. 1977;39(1):1-38. www.jstor.org/stable/2984875
2. Sterne JAC, White IR, Carlin JB, et al. Multiple imputation for missing data in epidemiological and clinical research: potential and pitfalls. *BMJ*. 2009;338:b2393. doi:10.1136/bmj.b2393
3. Harris CW, Kaiser HF. Oblique factor analytic solutions by orthogonal transformations. *Psychometrika*. 1964;29(4):347-362. doi:10.1007/BF02289601


© 2022 Barclay ME et al. *JAMA Health Forum.*

*eMethods 3 Table 1. Measures in the CMS Hospital Compare Star Ratings and corresponding domains, both according to the current Star Ratings and based on exploratory factor analysis.*

| Measure name | CMS (2021) Domain | Domain from exploratory factor analysis |
|---|---|---|
| Death rate for heart attack patients | Mortality | Mortality |
| Death rate for CABG surgery patients | Mortality | CABG outcomes |
| Death rate for COPD patients | Mortality | Mortality |
| Death rate for heart failure patients | Mortality | Mortality |
| Death rate for pneumonia patients | Mortality | Mortality |
| Death rate for stroke patients | Mortality | Mortality |
| Deaths among Patients with Serious Treatable Complications after Surgery | Mortality | Mortality |
| Central line-associated bloodstream infections (CLABSI) | Safety | Readmission |
| Catheter-associated urinary tract infections (CAUTI) | Safety | ED quality |
| Surgical site infections from colon surgery (SSI: Colon) | Safety | ED quality |
| Surgical site infections from abdominal hysterectomy (SSI: Hysterectomy) | Safety | ED quality |
| MRSA bloodstream infections | Safety | Readmission |
| C. diff bloodstream infections | Safety | ED quality |
| Rate of complications for hip/knee replacement patients | Safety | Readmission |
| Serious complications (PSI 90) | Safety | ED quality |
| Acute Myocardial Infarction (AMI) 30-Day Readmission Rate | Readmission | Readmission |
| Rate of readmission for CABG | Readmission | CABG outcomes |
| Rate of readmission for chronic obstructive pulmonary disease (COPD) patients | Readmission | Readmission |
| Heart failure (HF) 30-Day Readmission Rate | Readmission | Readmission |
| Rate of readmission after hip/knee replacement | Readmission | Readmission |
| Pneumonia (PN) 30-Day Readmission Rate | Readmission | Readmission |
| Rate of readmission after discharge from hospital (hospital-wide) | Readmission | Readmission |
| Rate of unplanned hospital visits after colonoscopy (per 1,000 colonoscopies) | Readmission | OP quality |
| Rate of inpatient admissions for patients receiving outpatient chemotherapy | Readmission | Readmission |
| Rate of emergency department (ED) visits for patients receiving outpatient chemo | Readmission | OP quality |
| Ratio of unplanned hospital visits after hospital outpatient surgery | Readmission | OP quality |
| Patients who reported that their nurses communicated well | Patient experience | Patient experience |
| Patients who reported that their doctors communicated well | Patient experience | Patient experience |
| Patients who reported that they received help as soon as they wanted | Patient experience | Patient experience |
| Patients who reported that staff explained about medicines before giving it to t | Patient experience | Patient experience |
| Cleanliness and quietness | Patient experience | Patient experience |
| Patients who reported that they were given information about what to do during t | Patient experience | Patient experience |
| Patients who understood their care when they left the hospital | Patient experience | Patient experience |
| Rating and recommendation | Patient experience | Patient experience |
| Percentage of healthcare workers given influenza vaccination | Timely and effective care | OP quality |
| Percentage of patients who left the emergency department before being seen | Timely and effective care | ED quality |
| Percentage of patients who came to the emergency department with stroke symptoms | Timely and effective care | ED quality |
| Percentage of patients receiving appropriate recommendation for follow-up screen | Timely and effective care | OP quality |
| Percentage of patients with history of polyps receiving follow-up colonoscopy in | Timely and effective care | OP quality |
| Percentage of mothers whose deliveries were scheduled too early (1-2 weeks early | Timely and effective care | OP quality |

© 2022 Barclay ME et al. *JAMA Health Forum.*

| | | |
|---|---|---|
| Percentage of patients who received appropriate care for severe sepsis and septi | Timely and effective care | ED quality |
| Percentage of outpatients with chest pain or possible heart attack who got drugs | Timely and effective care | ED quality |
| Percentage of patients receiving appropriate radiation therapy for cancer that h | Timely and effective care | OP quality |
| Average (median) time patients spent in the emergency department, after the doct | Timely and effective care | ED quality |
| Average (median) number of minutes before outpatients with chest pain or possibl | Timely and effective care | OP quality |
| Average (median) time patients spent in the emergency department before leaving | Timely and effective care | ED quality |
| % o/p with low-back pain who had MRI w/o trying recommended treatments first | Timely and effective care | OP quality |
| % o/p CT scans of the abdomen that were combination (double) scans | Timely and effective care | OP quality |
| % o/p who got cardiac imaging stress tests before low-risk outpatient surgery | Timely and effective care | Readmission |

© 2022 Barclay ME et al. *JAMA Health Forum.*

## eMethods 4. Technical details of the alternative approaches to domain weights and the implementation in the Monte Carlo simulations

The current CMS Hospital Compare approach combines seven domain scores using a weighted average. The four ‘outcome’ domains each receive a weight of 0.22 (i.e. totalling to a four-outcome domain weight of 0.88), and the single ‘process’ measure receives a weight of 0.12. This weighting convention evidently gives ‘outcome’ domains more weight than ‘process’ ones, reflecting stakeholder preferences, and is aligned with CMS quality initiatives, especially with the Hospital Value-Based Purchasing program.

As a plausible alternative approach, weights were drawn from appropriate log-normal distributions for which the mean value was the weight in current use. Let $wt_{domain}$ be the weight given to domain of quality $domain$ by the CMS Hospital Compare Star Ratings. For outcome measures $wt_{domain} = 0.22$, for process measures using the current Star Ratings groups $wt_{domain} = 0.12$, and for process measures using the alternate grouping based on factor analysis $wt_{domain} = 0.06$. The weights used in the Monte Carlo simulations, $mc\_wt_{domain,sim}$, were drawn from the distribution

$$mc\_wt_{domain,sim} \sim \text{expit}\left(\text{normal}(\text{logit}(wt_{doma}\quad), 1.5^2)\right)$$

This Monte Carlo simulation took the current weights as an appropriate starting point and then explored what happened if weights were varied around this starting point. To better appreciate this, it is worth considering the distribution on the ‘weight’ scale for each domain of quality in eMethods 4 Figure 1. Drawing from these distributions meant that 95% of weights for current outcome domains (mortality, readmission, safety of care, and patient experience) were between 0.05 and 0.81, while 95% of weights for current process domains (timeliness of care, efficient use of medical imaging, and effectiveness of care) were between 0.01 and 0.61. Across many simulations the drawn weights on average matched those used in the existing Hospital Compare Star Ratings.

Weight choice was independent for each domain. While in the current CMS approach, all outcome domains receive a weight of 0.22, the simulation was not restricted to give all outcome (or all process) domains the same weight. In the current CMS approach, the total weight across all domains adds up to 1. In the simulation, weights were rescaled so that the total of the weights was 1. For example, if by some unlikely fluke the drawn weight for each of the seven domains was 0.1, so that the total weight across all domains was 0.7, the weights would be upscaled by dividing by 0.7 so that the weight given to each domain when combining them would be around 0.14. This kept the composite score on a consistent scale between different Monte Carlo simulations.

© 2022 Barclay ME et al. *JAMA Health Forum.*

*eMethods 4 Figure 1. Distribution of domain weights used in the Monte Carlo simulation when the measures are grouped into the domains used in the current CMS Hospital Compare Star Ratings. The mean weight for the four outcome domains is 0.22 and the mean weight for the three process domains is 0.04, chosen to match those in the current specification. But there is a very large amount of uncertainty around what the correct weight should be.*

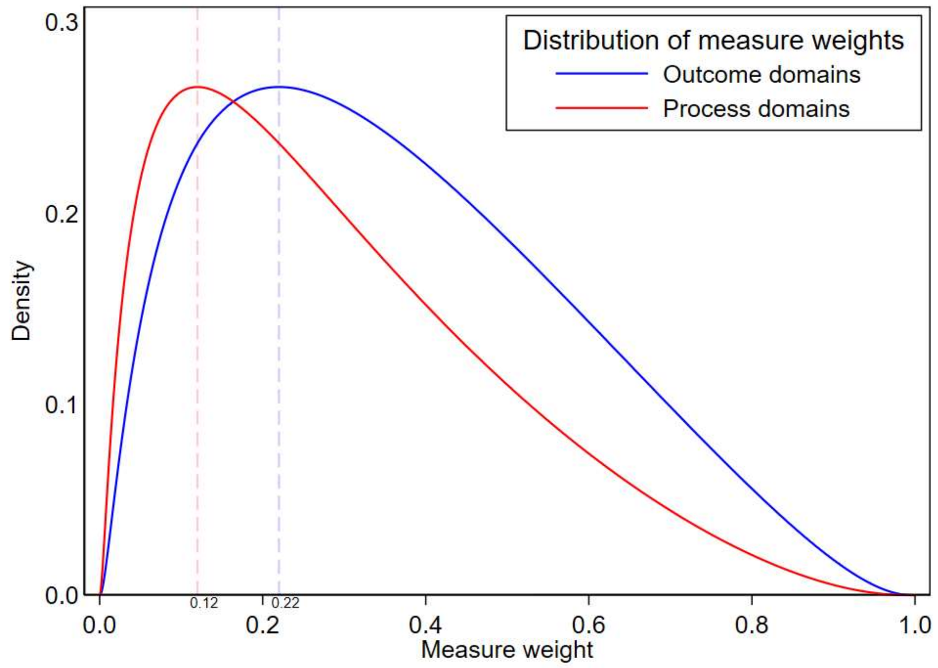


© 2022 Barclay ME et al. *JAMA Health Forum.*
